# The concept, influence, and mechanism of human work interruptions based on the grounded theory

**DOI:** 10.3389/fpsyg.2023.1044233

**Published:** 2023-02-16

**Authors:** Xiao Pan, Xiaokang Zhao, Huali Shen

**Affiliations:** ^1^Glorious Sun School of Business and Management, Donghua University, Shanghai, China; ^2^School of Business, Nantong Institute of Technology, Nantong, China

**Keywords:** human work interruptions, grounded theory, cognitive appraisals, affective responses, behavioral changes

## Abstract

With the development of mobile communication technology and the transformation of work methods and modes, work interruptions have become ubiquitous challenges for employees in the workplace. Less attention has been paid to work interruptions in China, especially the research on human work interruptions, which is different from virtual work interruptions. The present study carried out an in-depth interview with 29 employees. Based on the grounded theory method, a psychological and behavioral mechanism model of employees facing human work interruptions, namely, the “human work interruptions–cognitive appraisals–affective responses–behavioral changes” model, was constructed. It is found that (1) cognitive appraisals are the causes of different affective responses and behavioral changes of human work interruptions; (2) cognitive appraisals are feedback behaviors that refer to the reappraisals of the effectiveness and appropriateness of individuals’ affective responses and behavioral changes; and (3) personal traits and environmental characteristics at work influence the affective responses and behavioral changes of human work interruptions at the individual and organizational level. The model constructed in this study further extends the interruption theory and provides implications on how to process human work interruptions in human resource management practice.

## Introduction

It is easy to imagine the following scenario during our daily work: An employee is working on a document when an e-mail alert pops up on the computer screen. The employee might process the alert and continue working. Moments later, a colleague suddenly walks in and asks for help. The employee puts aside the paperwork in rogress and deals with requests for help from colleagues. According to a definition provided by Puranik et al. (2019), a work interruption is “an unexpected suspension of the behavioral performance of, or attentional focus from, an ongoing work task.” The unexpected suspension experienced by the employee above is a work interruption. With the development of mobile communication technology and the transformation of work methods and modes, work interruptions have become ubiquitous challenges for employees in the workplace (Sonnentag et al., 2018). In addition, due to the pandemic of COVID-19, a growing number of employees have started working from home. Blurring boundaries between work and family result in more frequent work interruptions (Leroy et al., 2021; Pan et al., 2022). According to a survey by Udemy (2019), about 75% of full-time employees of more than 1,000 U.S. office workers aged 18 or older admitted feeling interrupted during work hours. In addition, the researchers recorded about 80 interruptions in employees’ daily work and noted that uninterrupted task completion had evolved into a luxury for employees nowadays (Leroy and Glomb, 2018).

The important concept of work interruptions and related influence have attracted growing academic attention. Research shows that although some work interruptions only last a few minutes or less, the effects cannot be underestimated as they become more frequent and time-consuming (Chen and Karahanna, 2018). Some scholars believe that a person needs about 8 min of uninterrupted time to get involved in a state of creativity. When an employee is interrupted, it takes at least 25 min to adjust and return to the primary task. Work interruptions cause financial losses and impact employees’ performance, physical and mental health, and psychological reactions (Hunter et al., 2019; Ma et al., 2019). However, interruptions are not challenging as managers prioritize tasks and set deadlines and time slots to complete work tasks. Problem-solving thinking helps managers to develop coping skills and an absence of emotional intensity, and as a result, managers adopt a positive attitude toward work interruptions (Zoupanou, 2015). Many studies have investigated media-generated virtual work interruptions such as e-mails, alerts, notifications, and instant messaging (Sonnentag et al., 2018; Rosen et al., 2019). However, unlike virtual work interruptions, human work interruptions such as co-workers dropping in unannounced, providing updates, socializing, supervisors checking on work, or assigning new tasks are also widespread in many work environments. Such work interruptions often involve social norms, which may prevent knowledge employees from implementing various strategies to delay or circumvent the impact of interruption sources (Nardi and Whittaker, 2001). This research distills this kind of human-induced work interruptions into a new construct–human work interruptions. Considering the blurring boundaries between work and family due to the development of mobile communication technology, the transformation of work methods and modes, and the pandemic of COVID-19, human work interruptions occur both in and out of working hours. The current study defines human work interruptions as the unexpected interpersonal suspensions of the behavioral performance of, or attentional focuses from, ongoing work tasks in or out of working hours. There are similarities and differences between human work interruptions and virtual work interruptions. Human and virtual work interruptions both belong to work interruptions and have two necessary attributes: primary task suspension and unexpectedness. However, they have significant differences in terms of interaction channels, sensory cues, information richness, and intervention flexibility, which significantly impact interruption results. Therefore, a further understanding of human work interruptions has specific theoretical and practical implications. The experiment conducted by Nees and Fortna (2015) pointed out that human-initiated interruptions were more challenging to manage and control than computer-initiated interruptions under laboratory conditions. However, experimental data often depends on the laboratory environment and settings, and the causal relationship lacks authenticity in the workplace (Jenkins et al., 2016). Whether the laboratory results could apply to today’s complex workplace deserves further exploration and verification. Currently, there is few literature to explore the nature of human work interruptions and seek ways to manage them in the work environment.

Based on the above practical problems and theoretical gaps, this research attempts to adopt the grounded theory research method to explore and analyze core characteristics, consequences, and internal mechanisms of human work interruptions. The results can provide adequate theoretical support and management strategies for human work interruptions to ensure the sound and rapid development of employees and enterprises.

## Literature review

During the past 20 years, interruptions have attracted growing academic attention. However, the research is interdisciplinary and needs to be integrated. The proliferation and fragmentation of work interruptions research reveal the complexity of work interruption structure and also lead to the differences in the definitions and research methods of work interruptions, which hinder the further development of work interruption-related research. The literature review shows that the definitions of work interruption mainly focus on five attributes: suspension of primary tasks, unexpectedness, the existence of interrupting tasks, temporarily, and the source of interruptions (Couffe and Michael, 2017; Wilkes et al., 2018). Among them, the attributes of suspension of primary tasks and unexpectedness are necessary attributes that help define work interruption and distinguish it from other concepts. The attributes of the existence of interrupting tasks, temporarily, and the source of interruptions add unnecessary conditions to the definition (Puranik et al., 2019). Based on the above views, Puranik et al. (2019) have provided a new definition of work interruption, which is widely recognized and applied (Feldman and Greenway, 2021; Qiao et al., 2021).

Previous studies on work interruptions mainly focus on the negative impact on individual or group performance, including interrupted task errors, interrupting task errors, task delays, memory decline, and cognitive load (Marulanda-Carter and Jackson, 2012). Work interruptions are considered key impediments to individual performance at work (Perlow, 1999). Researchers often explain work interruption phenomenon through cognitive pathways and self-control pathways. With the development of human resource management, recent research increasingly pays attention to work interruptions’ impact on individual psychological outcomes. Frijda (1986) indicated that because work interruptions impacted work objectives, they might trigger affective responses. Zoupanou and Rydstedt (2019) found that affective rumination mediated and moderated the relationship between work interruptions and well-being. However, our understanding of how individuals feel and what are the related consequences when interrupted by human work interruptions are still incomplete. From a theoretical perspective, negative affect directly influences the development of employees’ individuals, teams, and even the whole company. Positive affect can promote not only the improvement of enterprise performance but also the generation of innovation. Affective mobilization and management of employees have become essential to enterprise management. Managers need to stimulate the positive affect of employees to bring their subjective initiative into full play and create more value for the enterprises. Hence, understanding whether and how work interruptions influence related affect is of growing importance. From a theoretical perspective, previous studies on work interruption events have emphasized the impact of negative affect, such as frustration, annoyance, and stress (Fletcher and Bedwell, 2016; Fletcher et al., 2018). However, some studies have found that when the interruption occurs at a more appropriate time, the interrupted important task is relatively simple, or the interrupted task has a strong correlation with the interrupted primary task, the negative affect will be relieved (Gluck et al., 2007; Puranik et al., 2019). Gasper (2018) also found some neutral affect associated with work interruption events recently. Furthermore, when work interruptions contribute to the achievement of the family goal or work goal, positive affect is activated (Hunter et al., 2019; Feldman and Greenway, 2021). It is these complex and inconsistent findings that inspire our research.

The affective event theory was proposed to explore the relationship among affective events, affective reactions, attitudes, and behaviors by organizational members at work (Weiss and Cropanzano, 1996). The theory points out that the affect caused by work events has a multidimensional structure and emphasizes the importance of studying the corresponding psychological experience. According to the theory, the structure of affective responses to events mainly includes mood and affect. Compared with mood, affect is more relevant to specific work events. If an employee was praised by the leader in the meeting, he would produce affective responses such as satisfaction, pleasure and excitement (Weiss and Cropanzano, 1996). Human work interruptions, as work events closely related to the work goal, will trigger the related affect, influencing work performance (Frijda, 1986). Mandler (1964) carried out the Experiment of interruptions in verbal sequences and proposed the interruption theory, which indicated that an interruption leads to an arousal state, followed by affect and behaviors. According to the two-factor theory of emotion proposed by Schachter and Singer (1962), the arousal state induced by an interruption may be accompanied by various affective responses, depending on the cognitive interpretation of the interruption (Morris and Perez, 1972). According to cognitive appraisal theory, the same physiological arousal state can trigger a variety of affect such as joy, anger, or jealousy, and the diversity of individual affect depends on their cognition of the situation (Lazarus and Folkman, 1984). Hence, the different affect and behaviors caused by interruptions in the previous studies might be explained by affective event theory and cognitive appraisal theory (Gluck et al., 2007; Fletcher et al., 2018; Gasper, 2018; Feldman and Greenway, 2021).

Previous studies have begun to focus on work interruptions and discuss the affect and behaviors they cause. However, research on human work interruptions is still scarce. This research adopts the grounded theory method to explore the concept, influence, and mechanism of human work interruptions. With the help of open coding, axial coding, and selective coding, the core category–“human work interruptions,” and the related consequences can be identified and summarized in a storyline. In addition, as a critical aspect of the grounded theory method, the maximum variation sampling allows the inclusion of ongoing participants of different age groups in the present study to build a theoretical framework (Boswell and Babchuk, 2022). This study mainly includes the following research contents:

1. Define the concept of human work interruption, and explore its core attributes, distinguishing it from the concept of virtual work interruption.
2. Explore the positive and negative dual affective responses and behavioral changes caused by human work interruptions.
3. Explore the mechanism of human work interruptions.

## Research design

### Methods

Grounded theory is a classical qualitative research method that deduces theoretical models by analyzing data coding, determining categories, clarifying storylines, and constantly revising theories until they are saturated (Glaser et al., 1968). Considering that the research on human work interruptions from the perspective of affect is still in the exploratory stage, the current study was conducted based on the grounded theory research method. Employees from multiple industries were recruited as interviewees for in-depth interviews. After that, a scientific research group consisting of the authors of the current study and one academic researcher conducted back-to-back coding analyzes based on the interview content. All of the researchers had management and psychology backgrounds. In open coding, the scientific research group captured the words and sentences initially used by the interviewees in the interviews, checked them word by word, identified the words and sentences highly related to human work interruptions, and obtained the initial categories. In axial coding, through theoretical guidance and literature references, the scientific research group condensed the initial categories and formed sub-categories. Then, the main categories were generalized by analyzing the relationships between sub-categories. Finally, the main categories were selectively coded according to the underlying internal logic and theoretical relations. At the same time, in order to ensure the reliability coding process, the following measures were taken in the coding process: Firstly, the data was processed entirely based on the interviewees’ description rather than guessing what they considered. Secondly, the scientific research group completed data coding together. When a researcher put forward a point of view, other researchers needed to question, verify, or supplement this point of view. This method reduced the one-sidedness of the conclusion caused by personal bias and ensured the consistency and integrity of coding results. Thirdly, adjustments and revisions were made according to third-party experts’ suggestions when controversial concepts or categories existed.

### Participants

In this study, about one-third of the participants were recruited from the interviewers’ social environment, including friends, relatives, and colleagues. It creates a basis of trust for honest answers to personal questions related to situations and true feelings (Lamnek and Krell, 2016). Participants were required to meet the following eligibility criteria: first, aged 18 or above; second, full-time employees; third, 1 year of tenure or above; fourth, be able to speak Mandarin; and fifth, have experiences of unexpected interpersonal suspensions of the behavioral performance of, or attentional focuses from, ongoing work tasks in or out of working hours. The initial 10 interviewees were from wholesale trade, retail trade, construction, manufacturing, service, communications, finance, public administration, and education industries. The other interviewees were selected randomly from the initial participants’ closer geographic environment, such as the workplace or social place, by adopting the snowball principle. Snowball sampling is a convenient sampling method for subjects with target characteristics. Individuals who had participated in the research helped to search for potential participants to expand the sample size (Naderifar et al., 2017; Zickar and Keith, 2023). Following the theory saturation principle of grounded theory, 29 participants (15 males and 14 females) were formally recruited in individual face-to-face interviews. The participants’ educational levels ranged from completing a bachelor’s degree to completing a doctorate degree. 69% had obtained master’s degrees or above. The ranges of participants’ age (Mean = 31.8, SD = 7.0) were 18–25 (28%), 26–35 (41%), and 36–45 (31%). A total of 59% participants were married, and among them, 45% participants had children living at home. For tenure, 38% participants had 1–3 years of working experience, 38% participants had 4–10 years of working experience, and 24% participants had been working for more than 10 years. The majority of the participants needed to work more than 40 h per week (83%). Thirteen industry types were involved, including one participant who worked in the wholesale trade industry, two participants who worked in the retail trade industry, one participant who worked in the agriculture industry, one participant who worked in the construction industry, four participants who worked in the manufacturing industry, two participants who worked in the transportation industry, three participants who worked in the communication industry, three participants who worked in the finance industry, one participant who worked in the insurance industry, one participant who worked in the real estate, three participants who worked in the services industry, three participants who worked in the public administration industry, and four participants who worked in the education industry. A total of 66% participants had worked for the private sector, and 24% participants had worked for the public sector.

### Data collection and processing

All interview procedures were approved by the authors’ University Ethics Committee and written informed consents were obtained from all participants. Eligible participants had 7 days to consider whether to take part in the interview. They could withdraw from the study at any time.

In order to deeply explore human work interruptions and related affective responses and behavioral changes, the research team conducted individual face-to-face semi-structured interviews in a quiet place, usually office and meeting rooms. A mock interview with three experienced employees who had worked for more than 5 years was carried out before the formal interview. The third question in the interview outline was revised to further inspire the interviewees’ deep thinking about human work interruptions according to their suggestions. During the interview, the interviewer first introduced the research purpose and the concept of human work interruptions. To avoid confusion, researchers explained human work interruptions thoroughly to Chinese employees and took examples to ensure the interviewees’ sufficient understanding of this literally translated concept. The interviewer would fully guarantee the data confidentiality and anonymity and ask the interviewee whether he could record with a recording device or write down with paper and pencil. After obtaining the interviewees’ written consent, the interviewer started to conduct interviews according to the interview outline. If deviations from the theme were found during the interview, the interviewees should correct them in time and guide them to return to the theme of human work interruptions and revisit the answers. The interviewees were encouraged to express all their thoughts on relevant issues thoroughly. Each interview lasted at least 30 min. All interviewees were able to express their opinions concerning human work interruptions thoroughly. The semi-structured interviews selected descriptive, evaluative, and contrast questions to ensure that interviewees understood question semantics. The interview outline was designed according to literature analysis and pre-interview results. The specific contents of the interview outline are shown in Table 1.

**Table 1:** Interview outline.

No.	Content
1	Please describe the human work interruptions experienced.
2	Please describe the consequences of human work interruptions.
3	Why are there different consequences when faced with human work interruptions?

After each interview, the research team sorted out the audio or text records in time. Nvivo12 Pro software was adopted to manage and analyze the data. The interview was terminated when the interviewees could not provide new information or repeated information began to appear. Two participants withdrew from the study. For the 29 data that met the requirements, 18 data was randomly selected as the original materials for coding. The other 11 reserved original materials were used for the saturation test of the results. On the basis of primary data, the accuracy of research data is improved through cross-verification by combining internal data and secondary data obtained from the website.

## Results

### Open coding

Three hundred seventy-one statements entered the open coding analysis program after repeated screening. After several back-to-back coding analyzes, the research team systematically integrated similar concepts. At the same time, some existing relevant literature was also referenced in the coding process, such as the segmentation of interruption sources, the comparison of cognitive appraisal dimensions, and the segmentation of work-related affect and behaviors (Reeve, 2001; Warr et al., 2014; Puranik et al., 2019). Finally, the research team obtained 36 initial categories.

### Axial coding

Axial coding further induced the initial categories obtained from open coding into sub-categories. For example, intervention priority, response priority, and attention priority in the initial categories were further induced into priority. The research team obtained 16 sub-categories based on 36 initial categories. Then, 6 main categories were generalized by analyzing the relationship between sub-categories. The main category, sub-category, and initial category are shown in Table 2.

**Table 2:** Human work interruptions axial coding.

Initial category	Sub-category	Main category
Suspension of behavioral performance of the task	Suspension	Human work interruption
Suspension of attentional focus from the task		
Inevitable	Unexpectedness	
Uncontrollable		
Intervention priority	Priority
Response priority
Attention priority
Abundant sensory channels	Richness
Information richness
Abundant social connection
Perceptibility	Personal relevance (Primary appraisal)	Cognitive appraisal
Effect degree		
Information coping ability	Coping ability (Secondary appraisal)
Social coping ability
No feeling	No affect	Affective response
Happy	Positive affect	
Excited		
Relaxed
Anxiety	Negative affect
Distress
Despair
Task completion speed	Task behavior change	Behavioral change
Task completion quality		
Language change	Social behavior change
Attitude change
Nervousness	Innate trait	Personal trait
Extroversion		
Agreeableness
Time management skills	Acquired trait
Multitask ability
Authoritarian leadership	Leadership style	Environmental characteristic
Humble leadership		
Relaxed and free	Colleague relations	
Serious and independent
Welfare and treatment	Resource allocation
Equipment and expenses

### Selective coding

In Selective coding, the core category emerged through the induction and refinement of the main categories. The research team established the relationships between the core category, main categories, and sub-categories after the emergence of the core category. Then, the storyline of “human work interruptions–cognitive appraisals–affective responses–behavioral changes” emerged. The interaction mechanism between the categories was analyzed to explore the path between human work interruptions and related affect and behaviors. Typical relational structures of main categories are shown in Table 3.

**Table 3:** Human work interruptions selective coding.

Generalization relationship	Relational structure	Connotation
Human work interruptions; cognitive appraisals; affective responses	Causality	The cognitive appraisals of human work interruptions determine the affective responses, but not the events themselves directly cause the affective responses. The cognitive appraisals of the events precede the affective responses. Through cognitive appraisals, employees judge whether individuals have enough resources to deal with human work interruptions to produce corresponding affective responses.
Affective responses; behavioral changes	Causality	Employees’ behaviors will be influenced by their affect, producing affect-driven behaviors and leading to behavioral changes.
Environmental characteristics; human work interruptions	Causality	Environmental characteristics can influence human work interruptions to trigger subsequent cognitive appraisals of employees. Environmental characteristics and work events are interchangeable.
Environmental characteristics; behavioral changes	Causality	Environmental characteristics directly influence employees’ behavioral changes.
Human work interruptions; cognitive appraisals; personal traits	Moderating relationship	Different personal traits can buffer the effects of human work interruptions on cognitive appraisals. Employees with positive affective traits are more likely to obtain positive cognitive appraisals during human work interruptions.
Affective responses; behavioral changes; coping abilities (secondary appraisals)	Causality	The coping abilities (secondary appraisals) also evaluate the effectiveness and suitability of individuals’ affective responses and behavioral changes, which are feedback behaviors.

### Construction of the conceptual model

**Figure 1:**
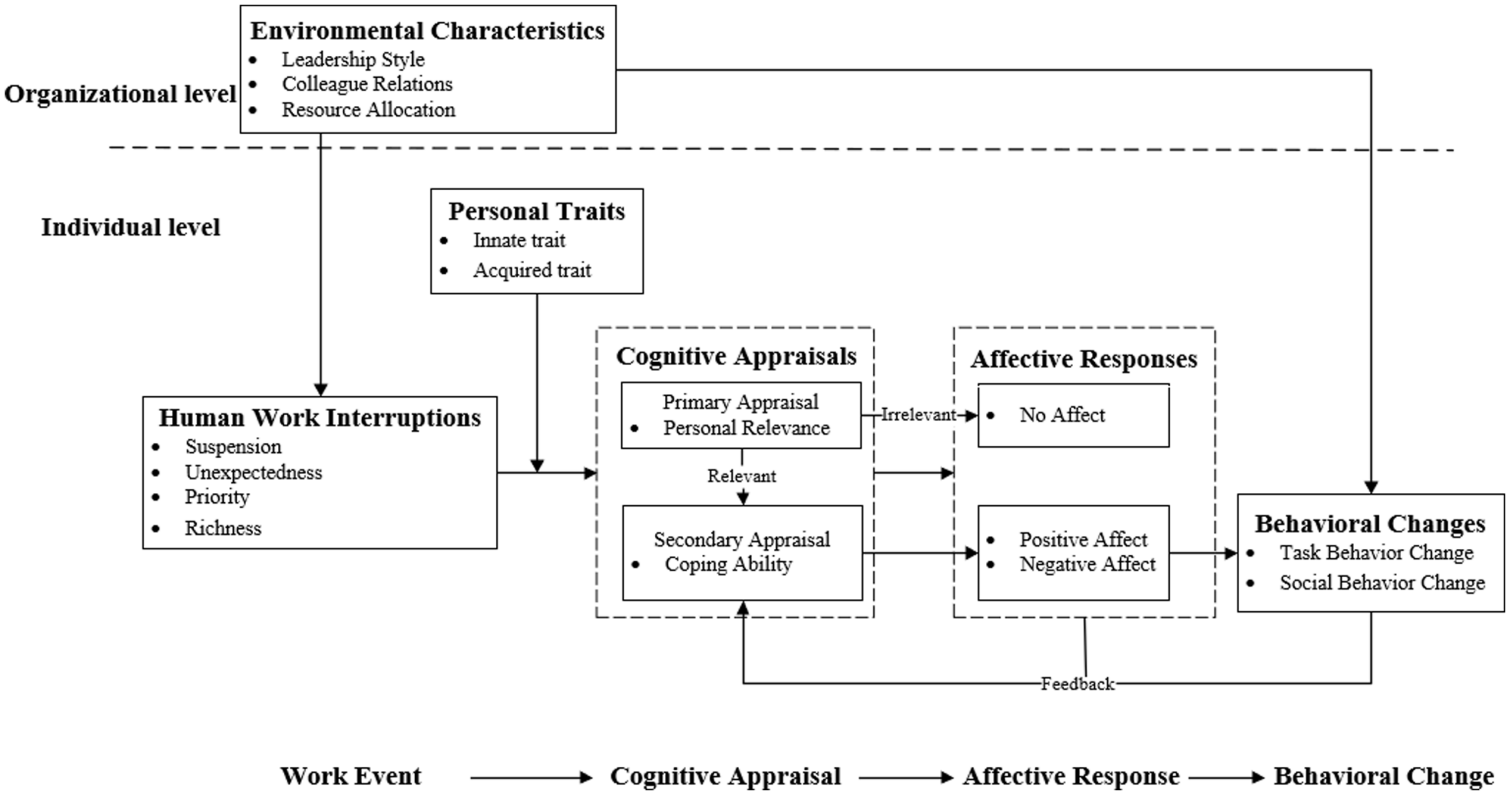
Human work interruptions–cognitive appraisals–affect responses–behavioral changes model.

Under the influence of environmental characteristics and personal traits, individuals’ cognitive appraisals of human work interruptions determine the affective responses, which leads to behavioral changes. Therefore, the “human work interruptions–cognitive appraisals–affective responses–behavioral changes” model constructed in this study is shown in Figure 1.

## Discussion

### The concept and attributes of human work interruptions

According to previous research, it is difficult to theoretically distinguish human work interruptions from virtual work interruptions from the relatively broad and inconsistent concept of work interruptions. The data in this study reflects four core attributes of human work interruptions: suspension, unexpectedness, priority, and richness. (1) Suspension refers to the suspension of behavioral performance of, or attentional focus from, an ongoing work task. (2) Unexpectedness refers to the unavoidable and controllable characteristics of the work events conducted by the interrupter. The two attributes above indicate that human work interruptions have the attributes of work interruptions and belong to work interruptions. (3) Priority refers to the fact that employees often fully understand the importance of interruptions by interrupters’ attitudes and descriptions. They try to predict, control, and coordinate such interruptions. They prioritize interruptions over ongoing tasks and deal with interrupters through dynamic face-to-face communication. (4) Richness refers to the fact that human work interruptions could stimulate the interrupted employee from visual, auditory, and even tactile channels to obtain rich information, which is more likely to trigger affective rumination and affective resonance, and establish deeper social connections. Because of the two core attributes of priority and richness, this research could distinguish human work interruptions from virtual work interruptions.

Previous research has found that the interruptions sources are the interruption stimulus or causes (Rivera-Rodriguez and Karsh, 2010). Various external interruption sources have been recorded in the workplace, such as device malfunctions, e-mails, phone calls, text messages, instant messaging chats, and face-to-face interactions (Nees and Fortna, 2015; Puranik et al., 2019). The distribution and frequency of these sources will vary according to the nature of the job and workplace. Equipment failures are more common interruptions in manufacturing setups for assembly line workers (Cai et al., 2017); phone calls and pagers are more common interruptions in medical institutions for doctors and nurses (Weigl et al., 2011); instant messaging and e-mails are more common interruptions in offices for knowledge workers (Fonner and Roloff, 2012). The concept and attributes of human work interruptions proposed in this study provide the basis for classifying work interruptions in various industries. The attributes of priority and richness indicate that human work interruptions are more challenging to avoid and control but are more likely to trigger affect and establish social connections.

### The mechanism of human work interruptions

#### The role of cognitive appraisals

According to the cognitive appraisal theory, appraisal refers to the process in which individuals constantly search for the required information and possible environmental threats, then carry out multi-round, uninterrupted evaluations of those stimulating events that are meaningful to them. The appraisal process includes primary appraisal and secondary appraisal. The secondary appraisal occurs after the primary appraisal (Lazarus and Folkman, 1984). The primary appraisal concerns two aspects. One is whether one is involved in an event personally, and the other is whether one has a stake in it. The involvement includes personal values, commitments, goals, and beliefs about oneself and the world. The stakes include concerns about a loved one and one’s physical and affective well-being (Lazarus and Folkman, 1984). In daily work environments, some mild events are unrelated to the individual’s goals and values. The appraisals of such events might only stay in the primary appraisal stages and often do not cause secondary appraisals. Comparatively, the second appraisal has a more meaningful analysis of the event, such as evaluating whether the individuals have enough resources to deal with the events (Huang et al., 2019). The results of this study show that when human work interruptions have impinged on no value, goal, needs, beliefs, or commitment, they will fall within the category of irrelevant; nothing will be lost or gained in the transactions. The cognitive appraisals of human work interruptions stay in the primary appraisal stage, and no affect is to be aroused in this process. When human work interruptions have been evaluated as relevant in the primary appraisal stage, the secondary appraisal stage will be aroused. Individuals will evaluate whether they have sufficient resources to cope with information and social interaction caused by human work interruptions in this stage. If the individuals are able to deal with the human work interruptions successfully, they will feel joy, satisfaction, and other positive affect. If the individuals are not able to deal with human work interruptions, they will feel pressure, anxiety, and other negative affect. The results are consistent with the affective event theory and cognitive appraisal theory (Lazarus and Folkman, 1984; Weiss and Cropanzano, 1996). Previous research has recorded positive or negative affect induced by work interruptions from colleagues and clients (Zoupanou, 2015; Feldman and Greenway, 2021). The results of this study indicate that employees engaged in healthcare, education, or administration seem to have more chance of being exposed to human work interruptions. Individuals’ evaluations of human work interruptions, more than the human work interruptions themselves, spark different affect (Fletcher and Bedwell, 2016). In addition, employees usually have multiple goals at one time, and the evaluations with different goals will trigger different affect in the appraisal processes (Boudreaux and Ozer, 2013). For example, suppose an employee engaged in Project B received a notice that the last Project A was approved and needed to be reviewed. In that case, he would evaluate whether the notice-receiving event was relevant to any of his goals. This event might be evaluated as positive for Project A and negative for Project B depending on the evaluations of achieving or hindering different goals. In the face of human work interruptions, employees might carry out multidimensional appraisals of information and social coping abilities and predict dominant affective responses and behavioral changes through comprehensive cognitive appraisal results.

#### The role of personal traits

Although numerous researchers have investigated the effects of work interruptions, only a few have considered the effects of buffering. According to affective event theory, personal traits are one kind of factor influencing affective responses (Weiss and Cropanzano, 1996). For instance, individuals with high positive affective traits seem to be more sensitive to positive affective stimuli (work interruptions), so they might produce more positive affective responses; The opposite is true for individuals with high negative affective traits. On the other hand, personal traits will directly influence employees’ cognitive appraisals of work interruptions, thus influencing their affect. Neuroticism, extraversion, and agreeableness are innate traits. Unlike innate traits, acquired traits, such as time management ability and multitasking ability, can be cultivated and reinforced as valuable and practical coping resources (Parke et al., 2018). The results of this study show that besides innate traits, acquired traits also buffer the relationship between human work interruptions and cognitive appraisals. These traits attenuate the negative indirect impact of human work interruptions on psychology and performance, facilitating goal attainment and task completion. Baethge et al. (2015) found that alertness moderated the relationship between interruptions and calmness. Pachler et al. (2018) found that trait polychronicity moderated the relationship between interruptions and job satisfaction. The results of this study are consistent with previous research.

#### The role of environmental characteristics at work

According to the affective event theory, environmental characteristics at work more or less influence affective experience through work events (Weiss and Cropanzano, 1996). Employees’ attitudes and behaviors are influenced by the work environment. One of the most used terms to describe the workplace environment is organizational climate. However, numerous studies have defined organizational climate as the common views of employees on organizational events and procedures. However, these perceptions represent individuals’ cognitive appraisals of the work environment from the individual level (Patterson et al., 2005). Numerous researchers have demonstrated relations between organizational climate and important indicators from different levels, such as organizational commitment, turnover intention, job satisfaction, individual job performance, and organizational performance in various industries. Among them, Chiang’s research showed that a depressive atmosphere positively correlates with emotional exhaustion at work in organizations (Chiang et al., 2020). Therefore, organizational climates could affect how employees think and feel about human work interruptions. The results of this study indicate that leadership styles, colleague relationships, and resource allocations influence human work interruptions as environmental characteristics at work. Compared with humble leaders, autocratic leaders tend to launch more human work interruptions. Close relationships among colleagues lead to more office “gossip” than cold ones. Such human work interruptions, while enhancing social connections, also negatively impact work progress. In addition, welfare and equipment expenses also impact employee satisfaction and change employees’ perceptions of human work interruptions.

#### Affective responses and behavioral changes caused by human work interruptions

Human work interruptions, as work events closely related to the work goals, will trigger the related affect, influencing work performances (Frijda, 1986). At the same time, affective event theory also focuses on the causes of the affective reactions of employees in the work environment and analyzes the mechanism of the affective reactions (Weiss and Cropanzano, 1996); That is, a cognitive appraisal is the necessary premise of the affective reaction. Employees’ cognitive appraisals of work events take precedence over affective reactions and determine affective reactions. Lazarus and Folkman (1984) proposed the importance of cognitive appraisal, advocated the existence of cognitive appraisal between an environmental stimulus and affective response, and established the most famous cognitive theoretical framework to date. At the same time, he emphasized that the nature of an individual’s idiosyncrasies and the interests of his specific environment determined his specific affect. Researchers have made great efforts to validate and expand the cognitive appraisal models (Luo and Chea, 2018). The results of this study showed that employees evaluated whether they had enough resources to deal with human work interruptions, which produced corresponding affective responses. Nevertheless, not all human work interruptions triggered affective responses (Zoupanou, 2015). Employees’ behaviors were influenced by affect, resulting in affect-driven behaviors, including task behavior changes and social behavior changes.

#### Feedback interventions

In organizational management, feedback is often used as a means of external intervention. Feedback such as praise from leaders, affirmation from customers, and encouragement from colleagues influenced individual efficacy, goals, coping styles, and related affect (van der Rijt et al., 2012; Li et al., 2014; Celuch et al., 2015). Individuals tended to search for the information needed in the environment and conduct multiple rounds of evaluation of meaningful events in real life (Lazarus, 2006). For example, an employee felt angry when he had evaluated a difficult and tedious task as a threat. The overt anger affected colleagues and also was noted and reacted to by its initiator. As a result, it might lead to guilt, fear, or other affect (Lazarus and Folkman, 1984). In instances of this feedback, the difficult and tedious task would no longer be assessed as a threat and might be reappraisal as unwarranted. According to the “human work interruptions–cognitive appraisals–affective responses–behavioral changes” model proposed in this study, the cognitive appraisals that occur in the coping process are the secondary appraisals after the primary appraisals. These processes also are seen as feedback behaviors that refer to the reappraisals of the effectiveness and appropriateness of individuals’ affective responses and behavioral changes. Through appraisals and reappraisals, individuals adjust their strategies to deal with threats and challenges caused by human work interruptions at any time and learn methods to cope with similar stimulating events. Ilgen et al. (1979) pointed out that a combination of feedback information, feedback recipients, and feedback deliverers influenced feedback behavior. Managers guided employees’ attention to task-related feedback and encouraged active communication between feedback recipients and feedback deliverers to improve feedback effectiveness (Whitaker and Levy, 2012; Mulder et al., 2013). Feedback management could trigger positive affect and behavioral changes upon the presence of work interruptions.

### Implications

The theoretical contribution of this study is embodied in the following three aspects. First, in recent years, the development of mobile communication technology and the transformation of work methods and modes have attracted growing academic attention to work interruptions (Foroughi et al., 2014; Keller et al., 2020). However, few studies on work interruptions, especially human work interruptions, have been conducted in China (Qiao et al., 2021). In the current background of China, this study combines existing literature with the grounded theory method to analyze human work interruptions. It extracts the “human work interruption” construct and its core attributes. Second, the “human work interruptions–cognitive appraisals–affective responses–behavioral changes” model proposed in this study indicates that cognitive appraisals determine different affective responses and behavioral changes when human work interruptions occur. This model is consistent with the affective event theory and the cognitive appraisal theory. Third, this study dissects the mechanism of human work interruptions at the individual and organizational level. As members of organizations, employees’ affect and behaviors cannot be separated from the dual influence of the individual and the organization. Previous studies on influencing factors have ignored the role of multiple factors at different levels (Lin et al., 2013; Parke et al., 2018; Puranik et al., 2019). This study establishes a more comprehensive theoretical framework by exploring how different factors, such as personal traits and environmental characteristics at work, influence the affective responses and behavioral changes of human work interruptions at the individual and organizational level.

This study proposes three practical implications: Firstly, managers could encourage employees to combine their own subjective perceptions of human work interruptions to make effective personal strategies through technical assistance and office layout upgrades to minimize the untimely potential human work interruptions (Nees and Fortna, 2015). Secondly, managers could integrate technical solutions with organizational practices. They could adopt human work interruption management and technology to arrange employees’ work schedules and make training plans. These methods can strengthen the benign communication between employees and train them how to interrupt others in a “calm” and “smart” way as much as possible, avoiding the generation of negative consequences. Thirdly, managers could change employees’ perceptions of human work interruptions from the conceptual level. Managers should emphasize rewarding employees’ innovative and cooperative behaviors instead of rewarding their working hours. It helps guide employees to positively evaluate human work interruptions, which inspires positive affective responses and behavioral changes.

### Limitations

There are still several limitations in the research. First, this study adopts the grounded theory research method to conduct coding analysis based on the interview content and secondary data. Although the research team conducts back-to-back coding, it still cannot altogether avoid the subjectivity of coding. Moreover, some concepts or categories might be omitted in the coding process, resulting in a loss. A further limitation is that the research team randomly selects individuals who have worked in China as samples and conducts the interviews. The data is collected only in Chinese. The specific characteristics of the Chinese language might limit this study’s generalization (Peng et al., 2019). Given that the effects of these cultures might vary, cross-cultural research would help us further understand the cultural influence on human work interruptions. Another possible limitation of our study is that grounded theory is a situational research method that could only obtain qualitative conclusions. Large-scale quantitative studies should be conducted to validate the psychological mechanisms of human work interruptions.

### Conclusion

This study aims to focus on a problem that exists in practice but has not caused theoretical concern – human work interruptions. Based on the grounded theory method, this study explores the concept, influence, and mechanism of human work interruptions to bridge this gap. This study further extends the interruption theory and provides opportunities to create changes in organizational practices. Managers could adopt technical assistance and upgrade office layout to improve environmental characteristics at work. By participating in regular training, employees could gain valuable personal traits and deal with human work interruptions in a positive way.

## Data availability statement

The datasets presented in this article are not readily available because confidentiality and anonymity were fully guaranteed in this manuscript. Requests to access the datasets should be directed to ✉ panx1222@163.com.

## Ethics statement

This study was approved by the Donghua University Ethics Committee and written informed consent was obtained from all participants. The patients/participants provided their written informed consent to participate in this study.

## Author contributions

XP was involved in data collection, data cleaning, theory building, writing, and data analysis. XZ helped in theory building, editing, project administration, and supervision. HS was responsible for data collection and editing. All authors contributed to the article and approved the submitted version.

## Funding

This work was supported by the Fundamental Research Funds for the Central Universities and Graduate Student Innovation Fund of Donghua University (Project No. CUSF-DH-D-2022055), Philosophy and Social Science Research in Colleges and Universities in Jiangsu Province (Project No. 2019SJA1478), and Fundamental Research Funds for the Central Universities (Project No. 21D110820).

## Conflict of interest

The authors declare that the research was conducted in the absence of any commercial or financial relationships that could be construed as a potential conflict of interest.

## Publisher’s note

All claims expressed in this article are solely those of the authors and do not necessarily represent those of their affiliated organizations, or those of the publisher, the editors and the reviewers. Any product that may be evaluated in this article, or claim that may be made by its manufacturer, is not guaranteed or endorsed by the publisher.
